# Characterization of Individualized Glycemic Excursions during a Standardized Bout of Hypoglycemia-Inducing Exercise and Subsequent Hypoglycemia Treatment—A Pilot Study

**DOI:** 10.3390/nu13114165

**Published:** 2021-11-21

**Authors:** Jan Brož, Matthew D. Campbell, Jana Urbanová, Marisa A. Nunes, Ludmila Brunerová, Dario Rahelić, Denisa Janíčková Žďárská, Arian Taniwall, Marek Brabec, Vojtěch Berka, Juraj Michalec, Jan Polák

**Affiliations:** 1 Department of Internal Medicine, Second Medical Faculty, Charles University, 150 00 Prague, Czech Republic; marisa_nunes.9@hotmail.com (M.A.N.); denisa.janickova.zdarska@email.cz (D.J.Ž.); ariantaniwall10@gmail.com (A.T.); vojtech.berka@seznam.cz (V.B.); jurajmich@gmail.com (J.M.); 2 School of Food Science and Nutrition, University of Leeds, Leeds LS2 9JT, UK; Matthew.Campbell@sunderland.ac.uk; 3 Department of Internal Medicine, Third Faculty of Medicine, University Hospital Královské Vinohrady, 106 00 Prague, Czech Republic; urbja@seznam.cz (J.U.); brunerova@seznam.cz (L.B.); 4 Vuk Vrhovac University Clinic for Diabetes, Endocrinology and Metabolic Diseases, Merkur University Hospital, 100 00 Zagreb, Croatia; dario.rahelic@gmail.com; 5 School of Medicine, University of Zagreb, 100 00 Zagreb, Croatia; 6 Faculty of Medicine, Josip Juraj Strossmayer University of Osijek, 310 00 Osijek, Croatia; 7 Institute of Computer Science, Academy of Science of the Czech Republic, 110 00 Prague, Czech Republic; mbrabec@cs.cas.cz; 8 Department of Pathophysiology, Third Faculty of Medicine, Charles University, 106 00 Prague, Czech Republic; polacisko@gmail.com

**Keywords:** type 1 diabetes, exercise, hypoglycemia, insulin therapy, glycemic excursion, hypoglycemia treatment

## Abstract

The glycemic response to ingested glucose for the treatment of hypoglycemia following exercise in type 1 diabetes patients has never been studied. Therefore, we aimed to characterize glucose dynamics during a standardized bout of hypoglycemia-inducing exercise and the subsequent hypoglycemia treatment with the oral ingestion of glucose. Ten male patients with type 1 diabetes performed a standardized bout of cycling exercise using an electrically braked ergometer at a target heart rate (THR) of 50% of the individual heart rate reserve, determined using the Karvonen equation. Exercise was terminated when hypoglycemia was reached, followed by immediate hypoglycemia treatment with the oral ingestion of 20 g of glucose. Arterialized blood glucose (ABG) levels were monitored at 5 min intervals during exercise and for 60 min during recovery. During exercise, ABG decreased at a mean rate of 0.11 ± 0.03 mmol/L·min^−1^ (minimum: 0.07, maximum: 0.17 mmol/L·min^−1^). During recovery, ABG increased at a mean rate of 0.13 ± 0.05 mmol/L·min^−1^ (minimum: 0.06, maximum: 0.19 mmol/L·min^−1^). Moreover, 20 g of glucose maintained recovery from hypoglycemia throughout the 60 min postexercise observation window.

## 1. Introduction 

The risk of hypoglycemia is substantially increased during exercise in type 1 diabetes patients [1]. This poses a significant challenge to diabetes self-management and remains a major barrier to participation in regular exercise [1,2].

The physiology of exercise-related hypoglycemia in diabetes has been reviewed in detail [3,4]. Below, we provide a short summary.

During a bout of moderate-intensity aerobic exercise (50–80%. VO_2_ max) in healthy individuals, several counter-regulatory mechanisms are gradually activated to maintain euglycemia. First, endogenous insulin secretion from the β-cells is suppressed to below fasting levels. The reduced insulin concentration enables the secretion of glucagon, which stimulates hepatic glucose output to match the rate of glucose uptake into the skeletal muscles. The decrease in insulin also sensitizes the liver to glucagon causing stimulation of glycogenolysis and gluconeogenesis. As the exercise bout progresses, other counter-regulatory hormones are released, including catecholamines, growth hormone, aldosterone, and cortisol, which stimulate hepatic glucose production and adipose tissue lipolysis, as well as inhibiting skeletal muscle glucose uptake, in order to protect against hypoglycemia. As exercise intensity increases above 60% of VO_2max_, lipid oxidation decreases, particularly in untrained individuals, and there is increased reliance on carbohydrates for energy provision [3,4].

In type 1 diabetes patients, the glucoregulatory response to moderate-intensity exercise is impaired, mainly due to the absence of the secretion of physiological amounts of endogenous insulin. Insulin is given artificially, but current therapeutic approaches are not able to precisely mimic physiological metabolic regulations. Consequently, circulating systemic insulin concentrations in type 1 diabetes patients may lead to relative hyperinsulinemia during and after exercise, promoting excessive glucose uptake and predisposing the patients to hypoglycemia. This is compounded by a dysregulated α-cell response, leading to lower glucagon levels and, hence, reduced hepatic glucose production. High concentrations of circulating insulin and skeletal muscle contractions exert additive effects on GLUT-4 translocation, resulting in heightened peripheral glucose uptake and further declines in glycemia, particularly in the postexercise period. Exercise-induced increases in muscle perfusion further increase insulin-mediated glucose disposal and, consequently, induce a drop in glycemia. Additional metabolic effects of higher insulin concentrations include the suppression of adipose tissue lipolysis and, therefore, increase fat oxidation in the skeletal muscle. These changes in fuel selection and oxidation alongside the imbalance between peripheral glucose disposal and hepatic glucose production eventually result in a significantly increased risk of hypoglycemia. The muscle glucose uptake remains increased for several hours as well as the muscle-enhanced insulin sensitivity. These changes may result in higher glucose uptake from blood to muscle and thus increase the risk of hypoglycemia in the postexercise period [3,4].

To prevent hypoglycemia during and after continuous moderate-intensity exercise, patients are typically advised to consume additional carbohydrates and reduce basal and/or bolus insulin doses/infusion rates [5,6,7,8]. However, even in patients treated with continuous subcutaneous insulin infusion (CSII), such changes to diabetes self-management require significant prior planning, which limits real-world applicability. In addition, current guidelines focus on fixed amounts of carbohydrates, which do not take into account glucose dynamics during exercise or recovery from exercise-induced hypoglycemia. Thus, hypoglycemia during exercise in type 1 diabetes patients remains both frequent and dangerous despite the application of current guidelines for hypoglycemia avoidance [3,9]. An intake of 15–20 g of glucose is generally recommended for nonsevere hypoglycemia treatment, irrespective of the particular hypoglycemia circumstances [10]. However, whether the glucose dynamics to ingested glucose for the treatment of hypoglycemia following exercise in type 1 diabetes patients is individualized has never been studied.

Therefore, in this study, we evaluated the glucose dynamics in individuals with type 1 diabetes during a standardized bout of hypoglycemia-inducing exercise and following the subsequent hypoglycemia treatment with oral carbohydrate administration.

We hypothesized that 20 g of glucose ingested after the bout of exercise would be individualized and would not be able to restore glycemia to the level above 3.9 mmol/L and keep it above this value for a period of 60 min after the end of exercise.

## 2. Material and Methods

### 2.1. Study Participants

Ten male adults with type 1 diabetes (mean ± SD: age 34.4 ± 3.9 years, diabetes duration 7.7 ± 1.7 years, BMI 23.4 ± 0.8 kg/m^2^, HbA1c 57 ± 4.8 mmol/mol (7.4 ± 0.62%) with undetectable levels of C-peptide participated in the study. Inclusion criteria were: otherwise healthy type 1 diabetes patients; an absence of diabetes complications and impaired awareness of hypoglycemia; the ability to follow the study protocol; and the performance of at least 1 h of physical activity three times a week. All participants were not taking any prescribed medications other than replacement insulin therapy via continuous subcutaneous insulin infusion. The clinical characteristics of the patients included in this study are shown in Supplemental Table S1.

### 2.2. Study Protocol

Following prescreening and enrollment, participants attended the laboratory at 08.00 a.m. on a single occasion after having a standardized breakfast (2 rolls, 50 g of ham, and 5 g of butter; total energy 1212 kJ; proteins 13.2 g, carbohydrates 41 g, and fat 7.9 g) at home at approximately 07.00 with an unaltered insulin dose. Visits to the laboratory were rescheduled in the event of a hypoglycemic episode such that no patient experienced severe hypoglycemia within 4 weeks and no mild hypoglycemia was experienced within 48 h of their laboratory visit. Furthermore, patients omitted participation in physical activity during the 48 h period prior to their experimental laboratory visit (stated by patients). Following arrival at the laboratory, patients assumed a seated rested position whilst placement of an indwelling catheter in the dorsal vein on the non-dominant hand was performed. The heated-hand procedure was performed with a warming blanket wrapped around the hand of the patient for the duration of the study [11]. At approximately 120 min post-breakfast (most of the food moves out of stomach [12]), participants performed a standardized bout of cycling exercise using an electrically braked ergometer (Ergoline 800, Ergoline GmbH, Bitz, Germany) at a target heart rate (THR) of 50% of the individual heart rate reserve, calculated using the Karvonen equation (THR = ((HRmax − HRrest) × %Intensity) + HRrest); HRmax was determined by subtracting age from 220 [13]. Exercise intensity was adjusted using a heart rate monitor (Polar Electro, Finland) to maintain THR. Exercise was interrupted when hypoglycemia occurred, defined as an ABG of ≤3.5 mmol/L with symptoms of hypoglycemia (mainly palpitations, shaking, drowsiness, sweating, hunger, incoordination, speech difficulty, and confusion [14]), or <3.0 mmol/L, irrespective of symptoms of hypoglycemia, or voluntary cessation during remarkable symptoms of hypoglycemia [15]. Upon achievement of hypoglycemia, exercise was terminated, and the patients received 20 g of glucose diluted in water (150 mL) for oral ingestion. ABG was continuously monitored at 5 min intervals during exercise and for up to 60 min postexercise. The ABG was measured immediately (Beckman Glucose Analyzer; Beckman Coulter Inc., Fullerton, CA, USA).

### 2.3. Statistical Analysis

A generalized additive model (GAM) [16] with smoothing spline [17] was applied to ABG data against time and was used to compute glucose concentration curves during exercise and 60 min after for each individual patient. Such elaborate statistical processing is necessary to overcome occasional data irregularity and discrete time spacing. The data analysis proceeded in three steps: (1) Fitted GAM was used to estimate glucose concentration on fine spaced time points (every 10 s from the start of the exercise until the end of the ABG measurements); (2) estimated ABG curves (penalized-likelihood-based estimates) were used to evaluate characteristics of interest (functionals such as maximal and minimal glycemic values and rate and duration of ABG changes); (3) characteristics from the previous point were summarized across the group, namely mean, median, standard deviation (SD), minimum (min), and maximum (max) to elucidate the extent of interindividual variability in various glycemia trajectory aspects. This approach allowed us to quantify not only the estimated glycemia trajectory but also its uncertainty (via asymptotic confidence intervals). We also fitted several regression models relating individual characteristics to age and BMI as obvious possible interindividual variability sources. All computations were in the R statistical environment, *R* [16].

## 3. Results

All participants completed the standardized bout of exercise but two patients did not complete the 60 min period recovery period due to personal reasons (they had to leave for unexpected duty) not related to the study; partial incorporation of their data was included in the final analysis as marked in Table 1. Each subject reached then maintained their individual target heart rate (range 120–137, mean 127 ± 5 beats per minute). Exercise was suspended in five patients due to eliciting glucose levels ≤3.5 mmol/L and in five patients experiencing remarkable symptoms of hypoglycemia at levels >4 mmol/L (mean ± SD 4.56 ± 0.36, range: 4.1 to 5.11 mmol/L). The mean exercise duration was 67.8 min (range: 27.8 to 108.8 min). The mean ABG value at the end of exercise was 3.63 ± 1.00 mmol/L (range: 2.45 to 5.11 mmol/L). The mean rate of ABG change during exercise was −0.11 ± 0.03 mmol/L.min^−1^ (range: −0.07 to −0.17 mmol/L.min^−1^).

**Figure 1:**
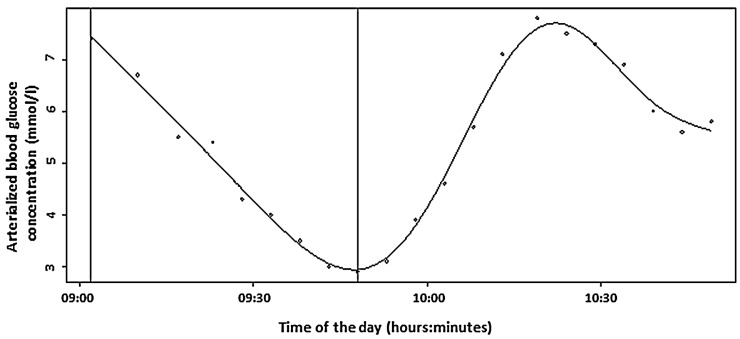
The curve of glucose concentration values during the study (Patient 1). The first vertical line represents the beginning of the exercise, and the second one represents the end of exercise and ingestion of 20 g of glucose.

**Figure 2:**
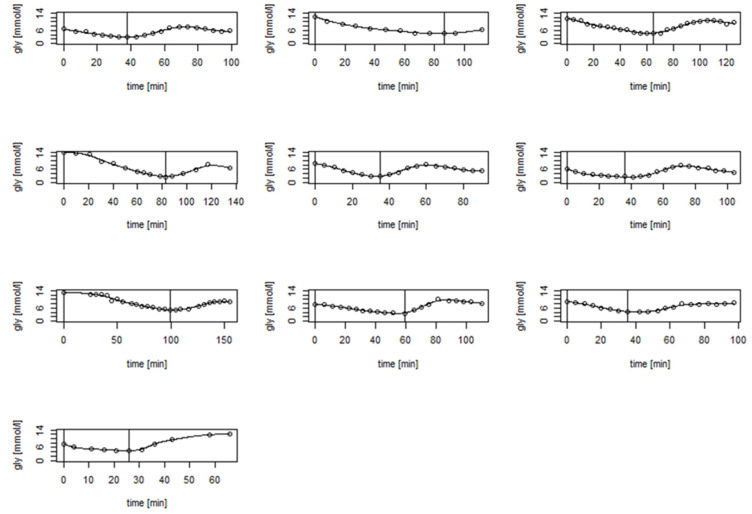
The curves of glucose concentration values of all participants during the study. The first vertical line represents the beginning of the exercise, and the second one represents the end of exercise and ingestion of 20 g of glucose.

During recovery, the mean rate of ABG increased after glucose ingestion was 0.14 ± 0.04 mmol/L.min^−1^ (range: 0.07 to 0.19 mmol/L.min^−1^). As such, ABG increased at a rate of 1 mmol/L per 16.5 ± 5.4 min (range: 9.2 to 25.8 min) on average. The maximal ABG level within 60 min after glucose ingestion was 8.62 ± 1.11 mmol/L (range: 7.70 to 10.60 mmol/L) and was reached on average 40.0 ± 9.9 min (range: 28.0 to 55.5 min) following cessation of exercise. An example of the curve of glucose concentration values is shown in Figure 1. The curves of each individual patient obtained during the study can be found in Figure 2 and in higher resolution in Supplemental Figure S1.

Regression models relating the duration of the BG increase from glucose ingestion to maximal glycemic value, the duration of the BG increase per the first 1 mol/l since glucose ingestion, and the rate of the BG increase since glucose ingestion until the maximal BG value to age, BMI, and weight were not significant.

## 4. Discussion

Understanding the dynamic nature of glycemia during exercise and recovery from exercise-induced hypoglycemia is paramount to effective type 1 diabetes self-management. As such, the aim of this study was to evaluate the speed of glycemic excursions during a defined level of hypoglycemia-inducing exercise and especially the rate of glucose recovery after oral hypoglycemia treatment.

To the best of our knowledge, this is the first study focused on such an important part of physical activity in type 1 diabetes patients. We showed that glucose concentrations decreased at a mean rate of 0.11 ± 0.028 mmol/L·min^−1^ during a steady state of cycling. However, we observed marked variability in glycemic responses both to exercise and following hypoglycemia treatment, with some individuals eliciting a glucose decline rate of 25% slower than the average, and other patients eliciting a more rapid glucose decline (~22–66% faster). Considering that trained individuals with type 1 diabetes typically show greater reductions in glycemia during aerobic exercise than individuals with reduced physical fitness [15], it is possible that the differences in the rates of glucose change in the present study may be due to differences in training status between patients. A further follow-up study assessing the contribution of training status and physical fitness on the rates of glucose change would help shed light on the possible mechanisms at play. Irrespective of the mechanisms at play, our results show that, if no usual hypoglycemia preventive steps were introduced, some patients could expect a drop of 3.3 mmol/L per a 45 min exercise session of the same intensity whilst other patients may encounter a fall in glycemia of 7.5 mmol/L during the same type of exercise session.

It is difficult to draw comparisons between our results related to exercise-induced falls in glycemia and other published studies as they differ in design, featuring different exercise modalities, intensities, and durations. Moreover, previous studies have typically fixed the duration of the exercise, whereas our study design intentionally guided patients to hypoglycemia [18]. Soo at al. employed a fixed bout 45 min ergometer-cycling session at a comparable intensity to that in our study in nine participants without carbohydrate supplementation [19]. The authors reported venous blood glucose changed during exercise in the range of −4.2 to 1.6 mmol/L (there was a glucose concentration elevation in some of the patients). We calculated the rate of blood glucose decrease. The mean rate of decrease was 0.026 mmol/L·min^−1^ (the patient with the largest glucose drop reached the rate of 0.093 mmol/L·min^−1^, similar to the average value found in our study), which is substantially higher than what we found in our study. A possible explanation for this difference is that the exercise sessions in the study of Soo at al. were performed in the morning after an overnight fast and before the subjects’ morning insulin dose; low concentrations of circulating insulin likely prevented rapid glycemia decreases, whereas higher circulating insulin concentrations, following the administration of prandial insulin in response to breakfast, likely accelerated the rate of decline in glycemia in our study. García-García et al. in a systematic review meta-analyzed 10 studies of various design focused on exercise in type 1 diabetes patients and found the rate of glycemia change between continuous exercise of moderate intensity and rest to be −0.073 mmol/L·min^−1^ (95 % confidence interval −0.10 to −0.05 mmol/L·min^−1^) [18]. These results seem to be partly concordant with the glycemia decrease rate we found, although the mean rate of blood glucose decrease calculated in our study was ~25% higher. This difference might be explained by high design variability of exercise type (walking, treadmill running, sprints, and ergometer cycling), intensity (20–65% VO_2max_), and duration (20–60 min) of the studies involved into the analysis, or the fasting/non-fasting states of the subjects performing the exercise.

A study focusing on glucose kinetics after glucose ingestion to treat hypoglycemia caused by exercise is not yet available. Thus, we consider this part of our investigation the most important one. The mean rate of glycemia increase we found was similar to that caused by insulin-triggered hypoglycemia. Slama et al. induced hypoglycemia by insulin injection and tested the rate of glycemia increase after ingestion of 15 g of glucose [20]. The mean glycemia increase 15 min after ingestion was 1.2 ± 0.4 mmol/L; in our study it was 1.0 ± 0.3 mmol/L. The rate of glycemia increase was very similar in seven patients of our study group, but in two patients the change was slower by about 40%, whereas in one patient it was faster by about 60%. We do not have a clear explanation for these differences as gastric emptying of liquids has generally been found to be unaffected by cycling exercise in type 1 diabetes patients at the exercise intensity used in our study [21,22]. There was no history and there were no symptoms of gastrointestinal autonomic neuropathy in any of the patients. Regression models relating the duration of BG increase from glucose ingestion to maximal glycemic value, the duration of BG increase per the first 1 mol/L since glucose ingestion, and the rate of BG increase since glucose ingestion until the maximal BG value to age, BMI, and weight were not significant. It is important to note that, while these variables were not detected as important players in this study, a much larger (multicenter) study would be needed when attempting to disprove their effect in modifying studied characteristics.

Eight patients completed the 60 min resting period after glucose ingestion. The maximal glucose level was reached after 40 min on average. In six patients, there was a decline in glucose concentration after the glucose peak, suggesting a possible risk of recurrent hypoglycemia. The key study message is that 20 g of glucose, contrary to our hypothesis, was able to maintain recovery from hypoglycemia in all study participants who reached glycemic values under 3.9 mmol/L and to then maintain glycemia above this limit for the period of 60 min after the end of exercise. The time of recovery among these subjects ranged from 9.2 to 24.2 min; the longest period was associated with the lowest glycemic value reached during cycling (2.5 mmol/L).

The strength of the study is that this is, to our best knowledge, the only study designed specifically to reveal the speed of blood glucose change during an exercise of a defined level leading to hypoglycemia and also to analyze glucose concentration kinetics after treatment of hypoglycemia caused by exercise. The intervals between points of blood glucose sampling are short, and arterialized blood was taken for analysis, thus allowing us to create a detailed picture of glucose concentration changes. The marked heterogeneity in glycemic responses both to exercise and following hypoglycemia treatment was observed. This suggests that individual evaluations of glycemic change during exercise might be required for each patient who wants to exercise to keep their physical activities person tailored and thus safer.

The study limitation is that, according to the study protocol, five patients terminated the exercise sessions close to but above the formal hypoglycemia levels due to the remarkable symptoms of hypoglycemia. Unfortunately, our study was limited by a tight budget that prevented, firstly, the collection of data from a higher number of participants, and secondly, an analysis of blood samples for cortisol, growth hormone, epinephrine, and in particular, insulin, all of which would have provided more robust results and a greater ability to explain the outcomes observed.

Regarding the number of participants, it should be noted that these types of studies, even those commonly cited, frequently involve a rather low number of subjects. For example, in the studies of Campbell et al. [6,7] ten and eight subjects were involved, while only nine subjects participated in the study of Soo et al. [19]. It should also be mentioned that our study participants showed great courage in “cycling to hypoglycemia” as this is an unpleasant and potentially dangerous event. Awareness of this fact, in addition to the demanding nature of the exercise itself, may contribute to the rather small number of participants typical to date for this type of study.

The baseline ABG levels differed among the participants as it is generally difficult for the patients to maintain a glycemic level close to the specific goal without an additional insulin dose or carbohydrate snack (this would influence the study results). These differences led to varying lengths of cycling (ranging from 27.8 to 108.8 min). To see the metabolic effect of these disparities, it would be necessary to monitor glycemia for a longer period of time (~up to 24 h), but this was not within the scope of our study.

We would like to conclude that, to our knowledge, our study is, at the moment, a unique one targeting the treatment of hypoglycemia connected to exercise. Regardless of its limitations, it presents valuable data showing “real lifelike” glycemic excursions during physical activity leading to hypoglycemia, and the same is shown after the hypoglycemia treatment. We also believe that the study, with its limitations, shows the hidden pitfalls and might, in a way, guide other teams focusing on this area. One of the pitfalls mentioned in the previous paragraph is the high variability of glycemia among the study participants at the beginning of the exercise. Inviting patients to stay overnight in the hospital before the study and using careful glycemia management could be a solution. Another pitfall is the hypoglycemia definition. We applied the same definition used by Al Khalifah et al. in their study [15], which allowed patients to terminate cycling due to hypoglycemia symptoms before the glycemia 3.5 mmol/L was reached. We also considered a definition based strictly on glycemic levels that would be more suitable for the study goals, but, as we wanted to make the study procedure easier for the patients, we finally chose the current one. We also took into account that hypoglycemia is unpleasant and is also a potentially life-threatening condition.

In any event, studies with higher numbers of participants are warranted to elucidate the effects of participants’ characteristics on glycemic excursions during exercise leading to hypoglycemia and hypoglycemia treatment.

## 5. Conclusions

We can conclude that a standardized bout of moderate-level hypoglycemia-inducing physical activity without previously provided preventive steps (decreased insulin dosing and extra portions of saccharides) led to hypoglycemia in all the participants. During exercise, the ABG minimal decrease was 0.7 mmol/L and the maximal was 1.7 mmol/L per 10 min. During recovery, after ingestion of 20 g of carbohydrates, the minimal ABG increase rate was 0.6 mmol/ and the maximal was 1.9 mmol per 10 min. Such an amount of glucose maintained recovery from hypoglycemia related to exercise in all study participants who reached glycemia under 3.9 mmol/L and kept glycemia above this value during the following resting period of 60 min.

## Figures and Tables

**Table 1:** Characteristics of blood glucose changes during and after exercise in individual patients.

Subject	BG at the Beginning of Exercise (mmol/L)	Rate of BG Decrease during Exercise (mmol/L·min^−1^)	Duration of BG Decrease until the End of Exercise (min)	BG at the End of Exercise (mmol/L)	Duration of BG) Increase per the First 1 mol/L Since Glucose Ingestion (min)	Rate of BG Increase Since Glucose Ingestion until the Maximal BG Value (mmol/L·min^−1^)	Glycemia Difference after 15 min Since Glucose Ingestion (mmol/L)	Duration of BG Increase Since Glucose Ingestion to Maximal Glycemic Value (min) ***	Maximal BG within 60 min after Glucose Ingestion (mmol/L) ***
1	7.4	0.10	45.3	2.9	14.5	0.14	1.03	34.8	7.7
2	14.0	0.10	93.0	4.6	25.8	0.06	0.58	28.0 *	6.4 *
3	11.8	0.10	72.0	4.6	13.2	0.14	1.14	44.0	10.6
4	14.2	0.12	93.8	2.6	13.0	0.14	1.15	36.7	7.7
5	9.1	0.17	38.7	2.7	9.2	0.19	1.60	28.0	7.9
6	7.2	0.10	47.7	2.5	24.2	0.15	0.62	33.7	7.9
7	13.2	0.07	108.8	5.1	18.2	0.08	0.82	53.2	9.2
8	8.2	0.08	63.2	3.5	16.0	0.18	0.94	34.0	9.8
9	9.9	0.12	47.5	4.1	14.2	0.07	1.06	55.5	8.2
10	8.6	0.14	27.8	4.4	14.7	0.18	1.02	42.2 **	12.0 **
Statistical characteristics									
Mean	10.57	0.107	67.8	3.63	16.5	0.136	0.996	40.0	8.62
Median	9.94	0.100	63.3	3.53	14.5	0.138	1.02	35.8	8.07
SD	2.79	0.028	25.5	1.00	5.4	0.042	0.291	9.9	1.11
Min	7.2	0.074	38.7	2.45	9.2	0.074	0.58	28.0	7.70
Max	14.2	0.166	108.8	5.11	25.8	0.186	1.60	55.5	10.60

BG = blood glucose; * patient completed only 28 min of the 60 min resting period; ** patient completed only 42.2 min of the 60 min resting period; *** data of the patients 2 and 10 who did not complete 60 min resting period after glucose ingestion were not involved to this analysis.

## Data Availability

The data presented in this study are available on request from the corresponding author.

## Supplementary Materials

The Supplementary Materials are available online at https://www.mdpi.com/article/10.3390/nu13114165/s1. Table S1: Participants’ clinical characteristics, Figure S1: Spline fits of individual patients.

## References

[1] Campbell M.D., Kime N., McKenna J. (2017). Exercise and physical activity in type 1 diabetes. Lancet Diabetes Endocrinol..

[2] Brazeau A.S., Rabasa-Lhoret R., Strychar I., Mircescu H. (2008). Barriers to physical activity among patients with type 1 diabetes. Diabetes Care.

[3] Scott S., Kempf P., Bally L., Stettler C. (2019). Carbohydrate Intake in the Context of Exercise in People with Type 1 Diabetes. Nutrients.

[4] Younk L.M., Mikeladze M., Tate D., Davis S.N. (2011). Exercise-related hypoglycemia in diabetes mellitus. Expert Rev. Endocrinol. Metab..

[5] Campbell M.D., Walker M., Trenell M.I., Jakovljevic D.G., Stevenson E.J., Bracken R.M., Bain S.C., West D.J. (2013). Large Pre-and Postexercise Rapid-Acting Insulin Reductions Preserves Glycemia and Prevents Early-but Not Late-Onset Hypoglycemia in Patients with Type 1 Diabetes. Diabetes Care..

[6] Campbell M.D., Walker M., Trenell M.I., Stevenson E.J., Turner D., Bracken R.M., Shaw J.A., West D.J. (2015). Insulin therapy and dietary adjustments to normalize glycaemia and prevent nocturnal hypoglycaemia after evening exercise in type 1 diabetes: A randomized controlled trial. BMJ Open Diabetes Res. Care.

[7] Campbell M.D., Walker M., Trenell M.I., Luzio S.C., Dunseath G., Tuner D., Bracken R.M., Bain S.C., Russell M., Stevenson E.J. (2014). Metabolic implications when employing heavy pre- and post-exercise rapid-acting insulin reduction to prevent hypoglycaemia in type 1 diabetes patients: A randomised clinical trial. PLoS ONE.

[8] Campbell M.D., Walker M., Trenell M.I., Stevenson E.J., Turner D., Bracken R.M., Shaw J.A., West D.J. (2014). A low glycemic index meal and bedtime snack prevents postprandial hyperglycemia and associated rises in inflammatory markers, providing protection from early but not late nocturnal hypoglycemia following evening exercise in type 1 diabetes patients. Diabetes Care..

[9] Adolfsson P., Mattsson S., Jendle J. (2015). Evaluation of glucose control when a new strategy of increased carbohydrate supply is implemented during prolonged physical exercise in type 1 diabetes. Eur. J. Appl. Physiol..

[10] Standards of Care (2021). Glycemic Targets: Standards of Medical Care in Diabetes—2021 American Diabetes Association. Diabetes Care.

[11] Van der Weerdt A.P., Klein L.J., Visser C.A., Visser F.C., Lammertsma A.A. (2002). Use of arterialised venous instead of arterial blood for measurement of myocardial glucose metabolism during euglycaemic-hyperinsulinaemic clamping. Eur. J. Nucl. Med. Mol. Imaging..

[12] Malmud L.S., Fisher R.S., Knight L.C., Rock E. (1982). Scintigraphic evaluation of gastric emptying. Semin Nucl. Med..

[13] Karvonen M.J., Kentala E., Mustala O. (1957). The effects of training on heart rate: A longitudinal study. Ann. Mcd. Exp. Bid. Fenn..

[14] McAulay V., Deary I.J., Frier B.M. (2001). Symptoms of hypoglycaemia in people with diabetes. Diabet. Med..

[15] Al Khalifah R.A., Suppère C., Haidar A., Rabasa-Lhoret R., Ladouceur M., Legault L. (2016). Association of aerobic fitness level with exercise-induced hypoglycaemia in Type 1 diabetes. Diabet. Med..

[16] Wood S.N. (2017). Generalized Additive Models: An Introduction with R.

[17] Eilers P.H.C., Marx B. (1996). Flexible smoothing with B-splines and penalties. Stat. Sci..

[18] García-García F., Kumareswaran K., Hovorka R., Hernando M.E. (2015). Quantifying the acute changes in glucose with exercise in type 1 diabetes: A systematic review and meta-analysis. Sports Med..

[19] Soo K., Furler S.M., Samaras K., Jenkins A.B., Campbell L.V., Chisholm D.J. (1996). Glycemic responses to exercise in IDDM after simple and complex carbohydrate supplementation. Diabetes Care.

[20] Slama G., Traynard P.Y., Desplanque N., Pudar H., Dhunputh I., Letanoux M., Bornet F.R., Tchobroutsky G. (1990). The search for an optimized treatment of hypoglycemia. Carbohydrates in tablets, solutin, or gel for the correction of insulin reactions. Arch. Intern. Med..

[21] Costill D.L., Saltin B. (1974). Factors limiting gastric emptying during rest and exercise. Appl. Physiol..

[22] Fordtran J.S., Saltin B. (1967). Gastric emptying intestinal absorption during prolonged severe exercise. J. Appl. Physiol..

