# Supplementary material for: Characterization of Individualized Glycemic Excursions during a Standardized Bout of Hypoglycemia-Inducing Exercise and Subsequent Hypoglycemia Treatment—A Pilot Study
Source: Nutrients. 2021 Nov 21;13(11):4165. doi: 10.3390/nu13114165 (PMC8619071; doi:10.3390/nu13114165)

1 Table S1 Participants´ clinical characteristics

Supplemental Table S1 Patients´clinical characteristics

Subject	Age (years)	DM duration (years)	BMI	HbA1c (mmol/mol)	Individual target heart rate (beats/ min)
1	45	8	24.6	58	125
2	40	7	24.3	53	130
3	36	9	22.5	62	137
4	35	9	22.7	51	120
5	38	5	24.1	60	124
6	40	7	22.1	61	127
7	39	8	22.8	56	124
8	38	6	23.6	49	132
9	42	11	23.4	63	126
10	31	7	23.9	57	127

2 Figures S1: Spline fits of individual patients

Units: Axis x: hours:minutes; Axis y: mmol/l

2.1 Patient 1

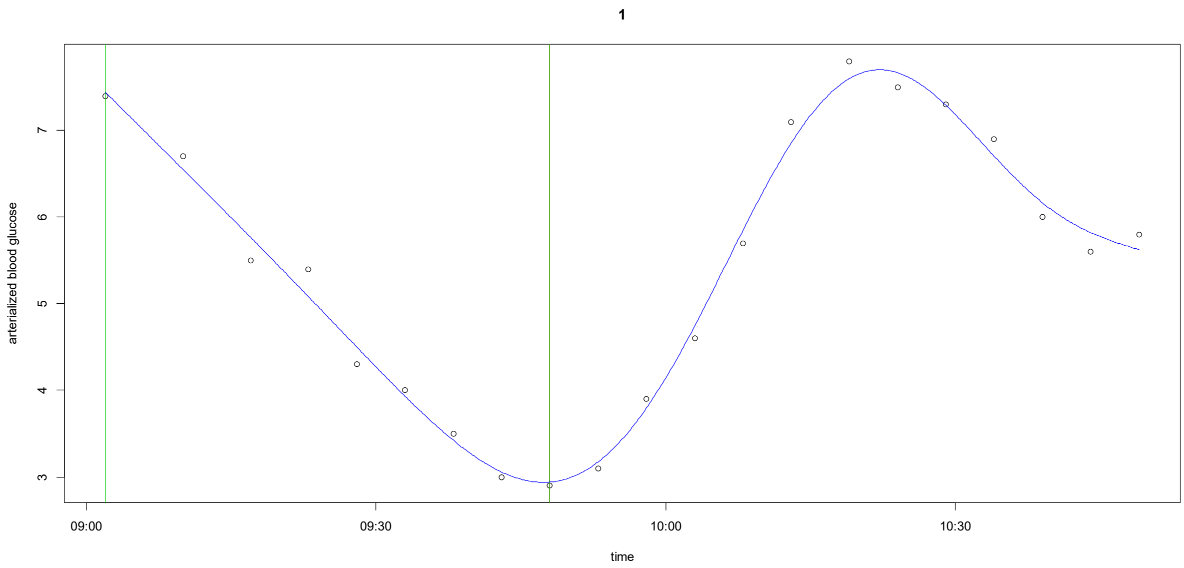

2.2 Patient 2

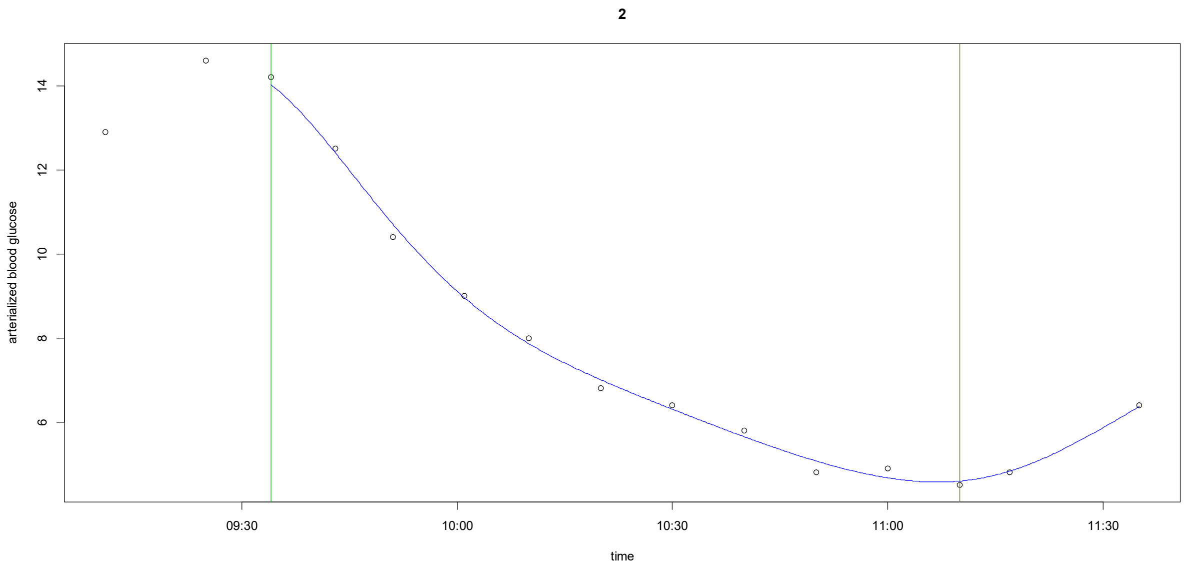

2.3 Patient 3

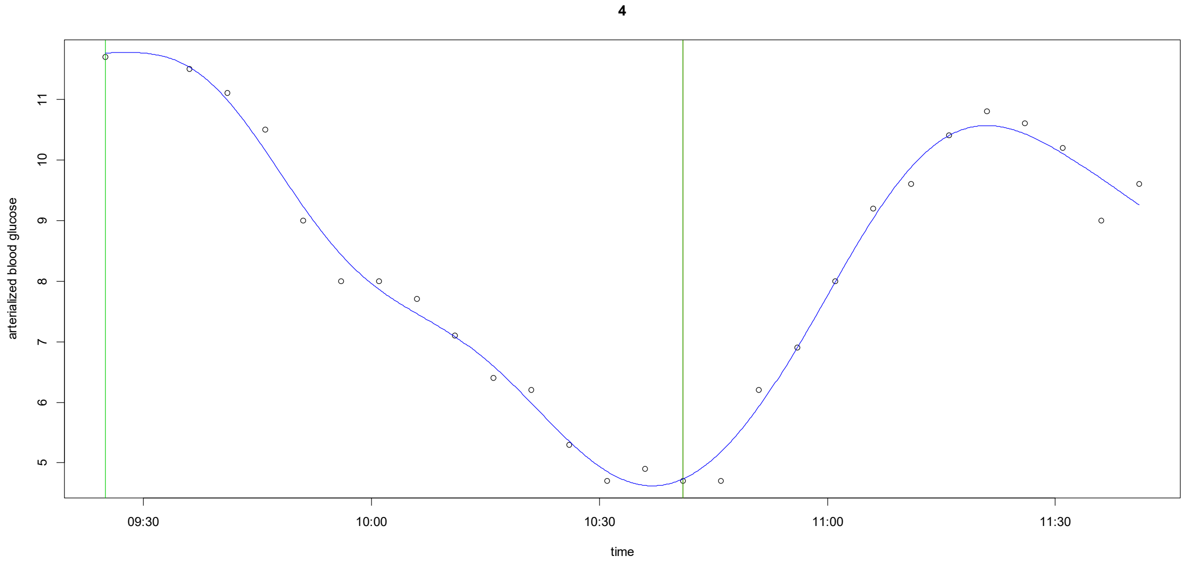

2.4 Patient 4

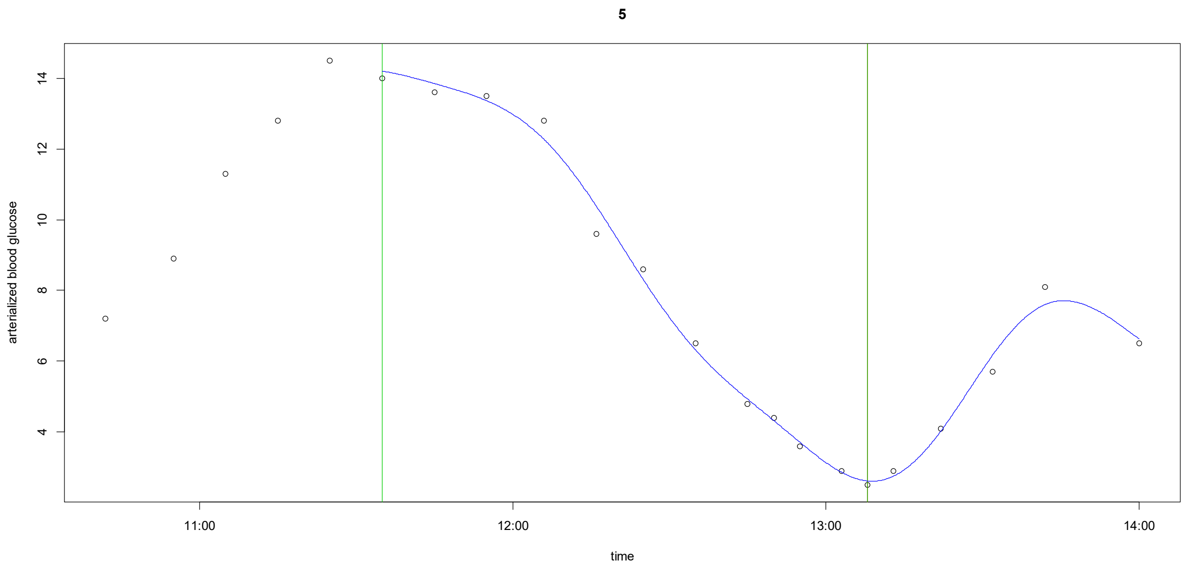

2.5 Patient 5

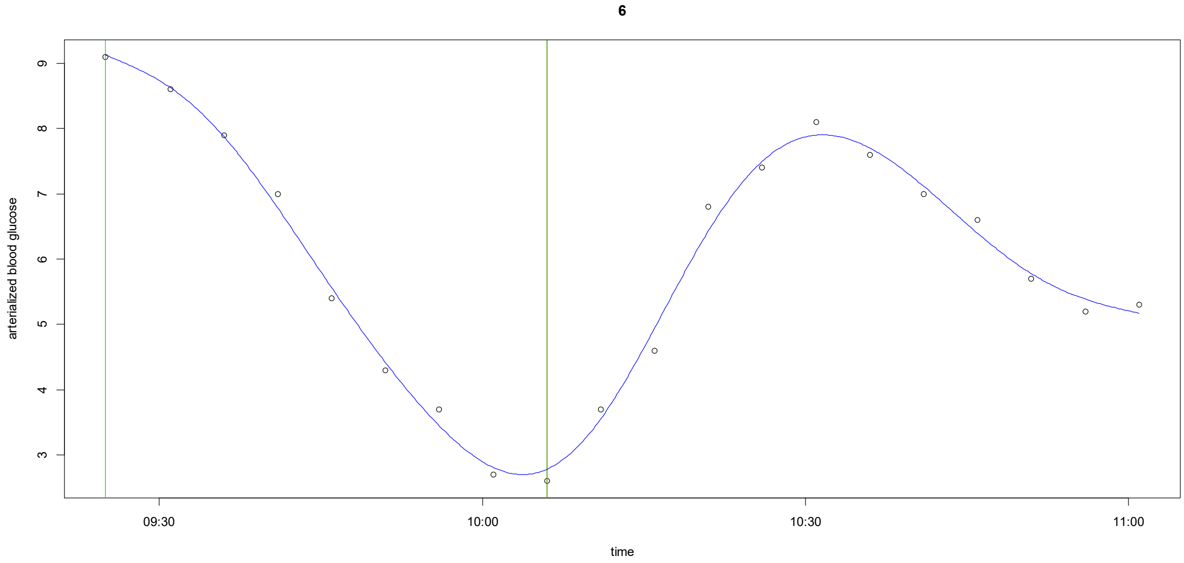

2.6 Patient 6

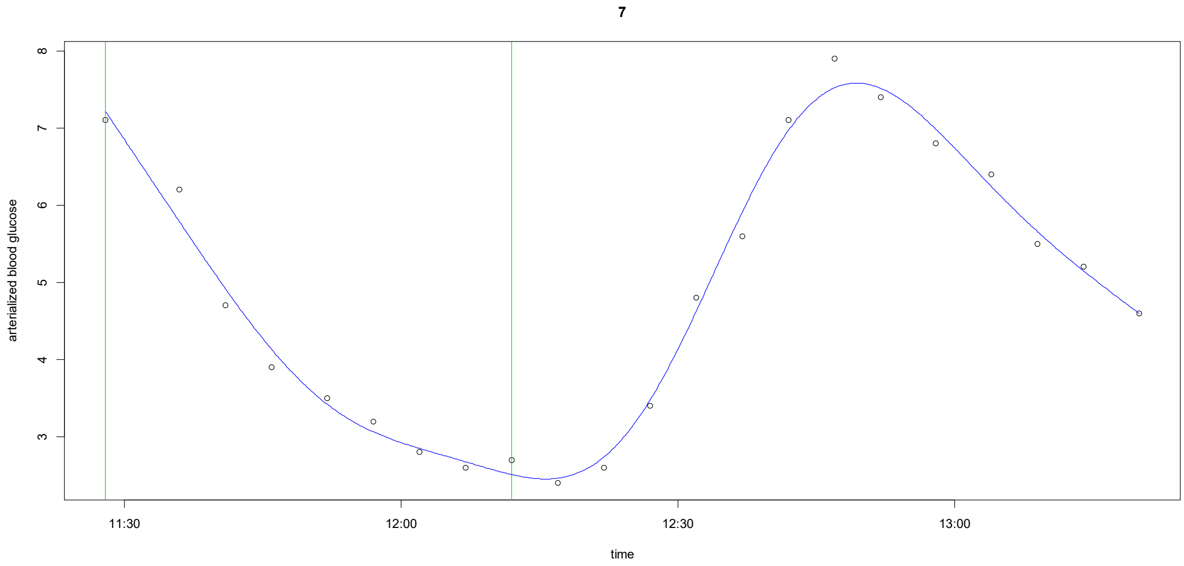

2.7 Patient 7

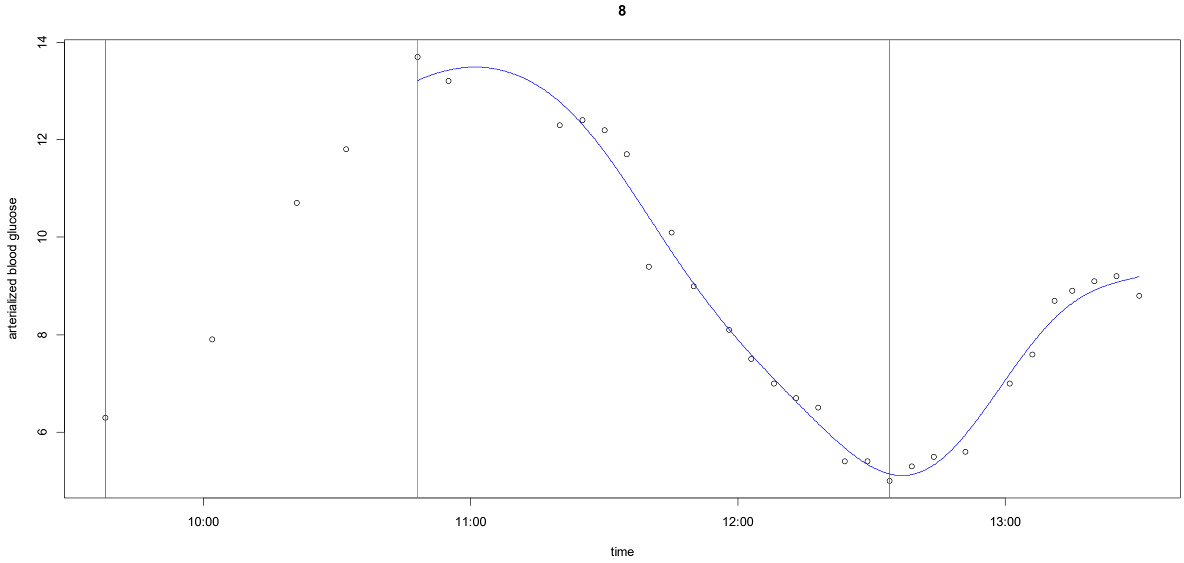

2.8 Patient 8

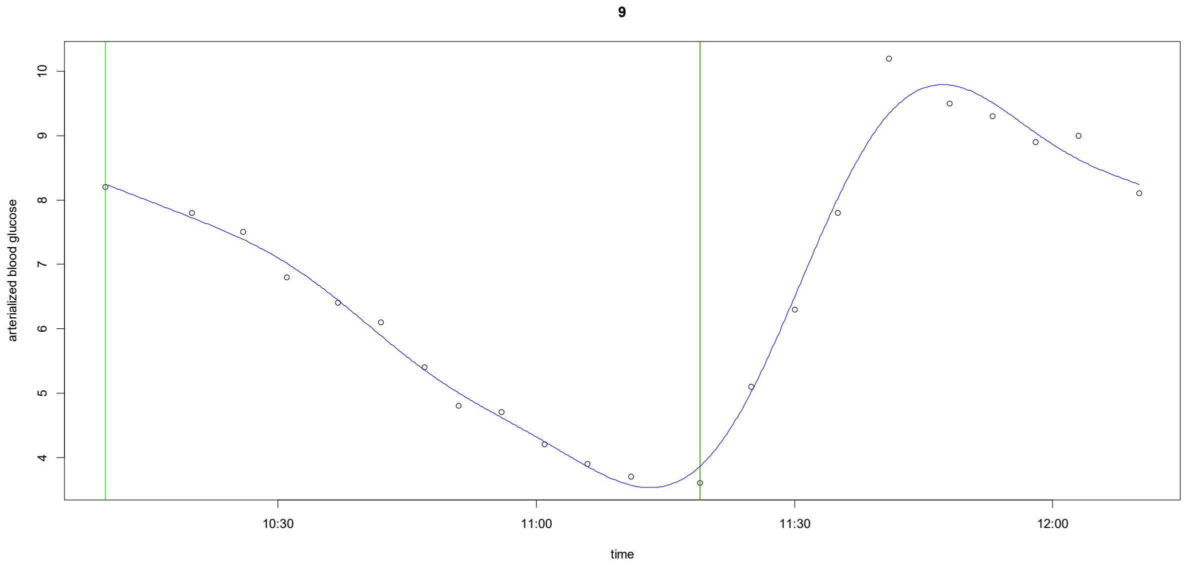

2.9 Patient 9

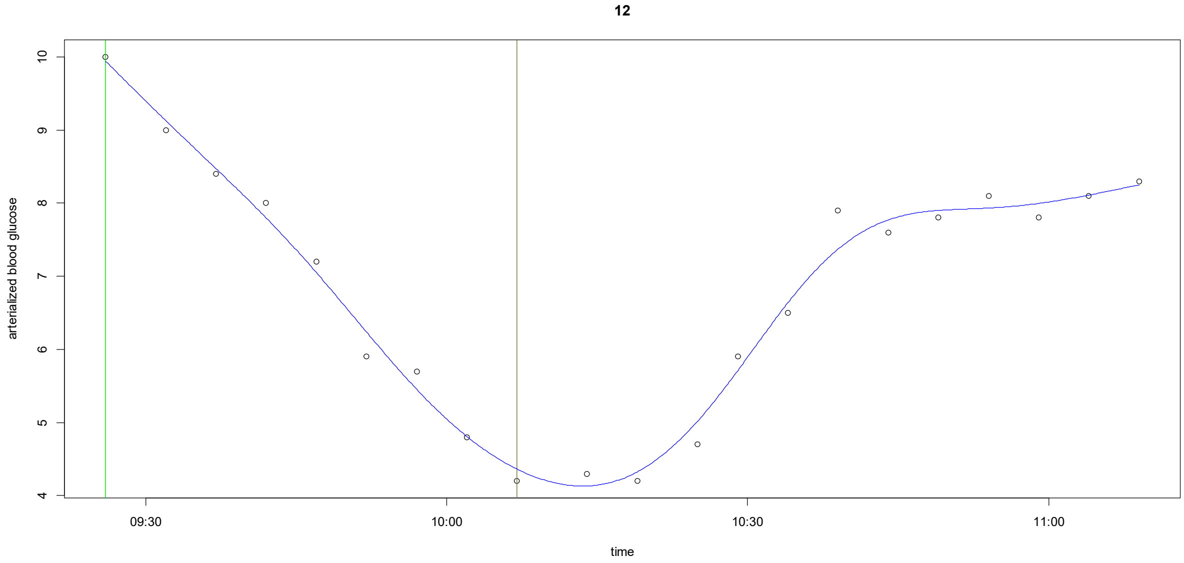

2.10 Patient 10

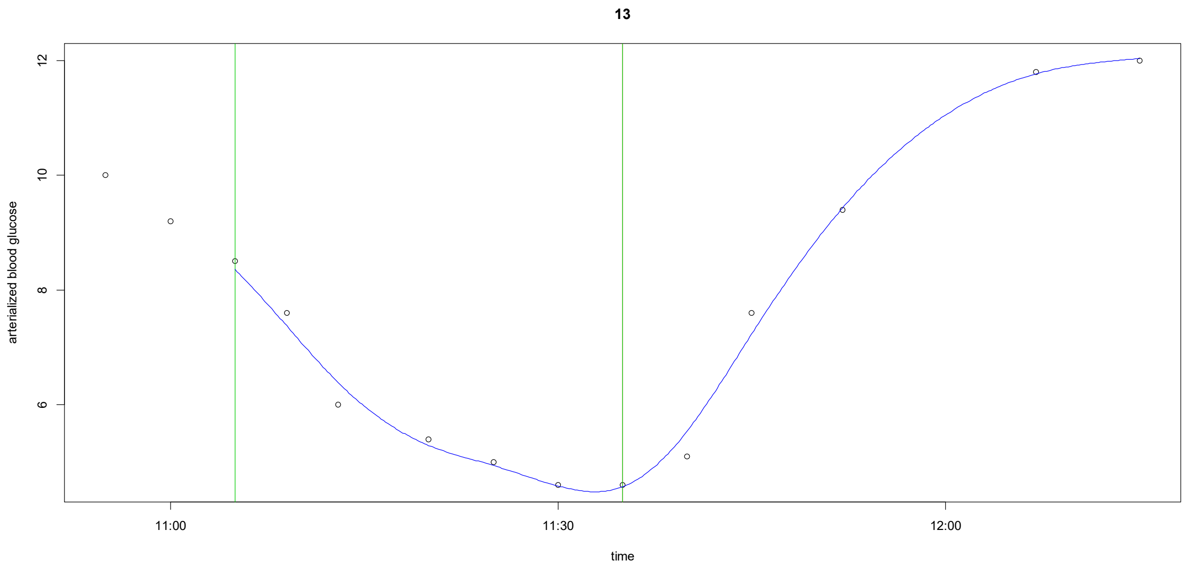

Supplement: Supplementary file 1 [file nutrients-13-04165-s001.zip › nutrients-1392047-supplementary.pdf]
